# Overloading the immunity of the mosquito *Anopheles gambiae* with multiple immune challenges

**DOI:** 10.1186/s13071-016-1491-8

**Published:** 2016-04-14

**Authors:** A. M. G. Barreaux, P. Barreaux, J. C. Koella

**Affiliations:** Laboratory of Ecology and Epidemiology of Parasites, Institute of Biology, University of Neuchâtel, Rue Emile-Argand 11, 2000 Neuchâtel, Switzerland

**Keywords:** Insect immunity, Immune overloading, Mosquito, Melanisation, *Anopheles gambiae*

## Abstract

**Background:**

Melanisation – the production and deposition of a layer of melanin that encapsulates many pathogens, including bacteria, filarial nematodes and malaria parasites is one of the main immune responses in mosquitoes. Can a high parasite load overload this immune response? If so, how is the melanisation response distributed among the individual parasites?

**Methods:**

We considered these questions with the mosquito *Anopheles gambiae* by inoculating individuals simultaneously with one, two or three negatively charged Sephadex beads, and estimating the melanisation as the darkness of the bead (which ranges from about 0 for unmelanised beads to 100 for the most melanised beads of our experiment).

**Results:**

As the number of beads increased, the average degree to which beads were melanised decreased from 71 to 50. While the darkness of the least melanised bead in a mosquito decreased from an average of 71 to 35, the darkness of the most strongly melanised one did not change with the number of beads.

**Conclusions:**

As the number of beads increased, the mosquito’s immune response became overloaded. The mosquito’s response was to prioritise the melanisation of one bead rather than distributing its response over all beads. Such immune overloading may be an important factor underlying the evolution of resistance against vector-borne diseases.

**Electronic supplementary material:**

The online version of this article (doi:10.1186/s13071-016-1491-8) contains supplementary material, which is available to authorized users.

## Background

Immune responses are complex pathways that can kill invading pathogens and thus protect individuals from harmful infections. Many parasites have therefore evolved mechanisms to avoid the immune response by, for example, hiding within cells [1, 2], switching their surface antigens to prevent recognition [1–5], or actively suppressing the response [1, 6, 7].

Alternatively, the immune response may simply be overloaded by the immune challenge. In other words, if the infectious dose is high enough, the immune response might no longer cope with all of the parasites. Although this possibility appears to be generally overlooked, it finds support, for example, by experimental data suggesting that the immune melanisation response of honey bees is limited by their ability to replenish the phenoloxidase reserves needed for melanisation [8].

An overloaded immune system would be visible as an outcome lying between two extremes. At one extreme, few parasites could be targeted and dealt with optimally, while the remaining ones cannot be dealt with. At the other extreme, all parasites could be dealt with similar, but weak efficacy.

We considered these possibilities with the melanisation immune response of the mosquito *Anopheles gambiae* [9]. In mosquitoes, this immune response is effective against bacteria [10], filarial nematodes [11] and, in some cases, *Plasmodium* [12, 13].

A standard tool to study the melanisation response is to inject a small bead into the thorax and to measure the degree to which it is encapsulated with melanin [10]. While in many studies [11–13], only one bead is injected, we studied the potential immune overloading by investigating the degree to which a mosquito could melanise one, two or three beads injected into its thorax.

Beads are useful in this context, for our aim was to investigate the direct effect of immune stimulation on the immune response, without having to deal with the complicating effects of an invading pathogen such as pathogenicity or immune-suppression, which may be linked to pathogen load. Furthermore, we considered only the melanisation response, as it is difficult to evaluate the efficacy of the immune system against dead bacteria (and, again, we did not wish to consider a living pathogen like Lambrechts et al. [14]).

## Methods

The experiment was performed at 26 ± 1 °C, 70 ± 5 % relative humidity and a 12:12 h light: dark cycle. We used the Kisumu laboratory strain of *An. gambiae* originating from western Kenya [15]. We selected newly hatched larvae haphazardly and reared them individually in 12-well-plates filled with 3 ml of deionised water to which we added Tetramin^TM^ baby fish food daily (day of hatching: 0.04 mg per larva; 1 day old: 0.06 mg; 2 days old: 0.08 mg; 3 days old: 0.16 mg; 4 days old: 0.32 mg, 5 days old or older: 0.6 mg) [16]. Each pupa was put into a 180 ml plastic cup covered with mosquito netting. After emergence, males were removed and females were given access to 10 % sugar solution*.*

Two days after emergence, 60 females were chilled on ice. We inoculated each female with 1, 2 or 3 negatively charged Sephadex CM C-25 beads (40–120 μm in diameter, Sigma-Aldrich, Steinheim, Germany), injected simultaneously together with 0.1 μl sterile saline solution (1.3 mM NaCl, 0.5 mM KCl, 0.2 mM CaCl_2_ [pH 6.8]) into the left side of the thoracic cavity [6, 17]. We added 0.001 % methyl green to the saline to help us see the transparent beads. After inoculation, mosquitoes were returned to their cups and given 10 % sugar solution.

Two days later, i.e. 24 hours after the melanisation of a single bead has reached its plateau [12], we dissected the mosquitoes that were alive to measure their wing length (as a proxy of body size), the size of the beads and the degree of their melanisation.

The mosquitoe’s wings were removed, fixed onto slides and measured from the tip to the distal end of the alula (excluding the fringe) with the software ImageJ (version 1.47f7). We used the mean length of the two wings in our analyses. On a glass microscope slide we then separated the thorax from the head and from the abdomen using forceps in the same saline solution used for injection. We opened the thorax with forceps, retrieved the beads and put them onto a slide in solution to take the picture. In this, and previous experiments, the beads were found in the thorax and had not moved to the abdomen. When we found several beads, they were not in contact with each other. Most beads were floating freely in the haemocoel, but some had tissues attached. In this and other experiments, we did not find any effect of the presence of tissue to bead melanisation. We took a digital image of each bead at a standard lighting setting with a microscope (Olympus® BX 50 equipped with a CC-12, Soft Imaging System), and measured the cross-sectional area of each bead and its mean grey value with the software ImageJ. The grey-value was standardized by linear interpolation to a value between 0 (which corresponded to the grey value of an unmelanised bead) and 100 (corresponding to a heavily melanised bead).

We tested the data for normality with Shapiro tests and for homoscedasticity of the variance with Bartlett tests. As initial analyses of full models with interactions terms and models without non-significant interactions showed that the size of beads had no influence on the conclusions, we omitted bead size from the analyses shown here.

To assay whether the number of beads influenced the melanisation response, we analysed the melanisation of each bead with a linear mixed model (function lmer) that included the number of injected beads and wing length as independent variables and the mosquito as a random factor. In a second analysis, we assayed the variability of the melanisation response as a function of the number of beads. As there is no variability in mosquitoes inoculated with one bead, we could not perform a standard assay of repeatability. Therefore, we analysed the bead with the highest melanisation or the lowest melanisation in each mosquito with an ANCOVA, again including the number of injected beads and wing length as independent variables. We analysed the survival rate two days after injection with a binomial GLM including the number of beads inoculated and wing length as independent variables. All analyses were performed with R version 3.0.2.

## Results

Of the 60 mosquitoes we had inoculated, 44 were analysed (18, 12 and 14 mosquitoes inoculated with 1 bead, 2 beads or 3 beads, respectively). This difference in survival was statistically not significant (Chi-square = 5.21, *P*  = 0.076).

**Fig. 1:**
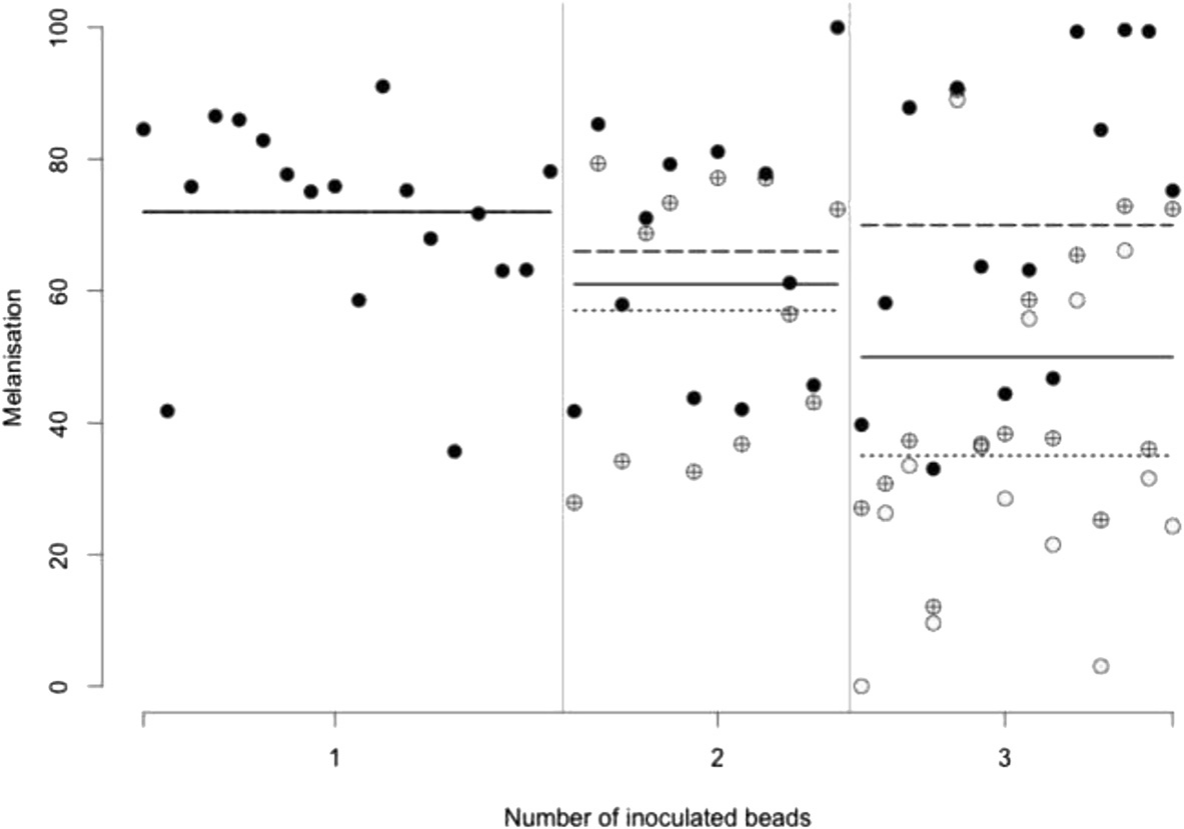
Bead melanisation as a function of the number of inoculated beads. Each point shows the melanisation of a single bead (with values ranging from 0 for non-melanised beads to 100 for the heavily melanised ones). The solid points represent the highest melanisation value per mosquito, the crossed points represent the intermediate melanisation value and the empty points represent the lowest melanisation value. The solid lines represent the mean melanisation per bead treatment, the dashed lines the mean of the highest melanisation and the dotted lines the mean of the lowest melanisation

The mean level of melanisation decreased from 71 (± sd = 14) in mosquitoes inoculated with one bead, to 50 (± sd = 27) in those inoculated with three beads (Fig. 1; Chi-square = 8.47, *P* = 0.01). There was no effect of wing length (Chi-square = 0.106; *P* = 0.74) or the interaction of number of beads and wing length (Chi-square = 1.267; *P* = 0.53).

While the highest melanisation per mosquito was independent of the number of beads (69 ± sd = 19; *F* = 0.380, *P* = 0.686); the lowest melanisation decreased from 71 (± sd = 14) in mosquitoes inoculated with one bead, to 35 (± sd = 25) in mosquitoes inoculated with three (Fig. 1; *F* = 12.76; *P* < 0.001). Neither highest (*F* = 0.376, *P* = 0.543) nor lowest (*F* = 0.13, *P* = 0.72) melanisation were affected by wing length.

## Discussion

Our data suggests that the mosquitoe’s melanisation immune response becomes overloaded by a small number of injected Sephadex beads. When the immune response is overloaded, the strength of melanisation is not distributed uniformly among the beads, but one bead is prioritised to the detriment of the immune response against others. Indeed, almost all mosquitoes were able to melanise one bead to a high degree, whether it had been inoculated with one, two, or three beads, but additional beads were melanised much less. The least-melanised bead in each mosquito inoculated with three beads was melanised only about half as much as the best one.

As survival differed (though non-significantly) among the three groups of mosquitoes, we analysed a non-random subset of mosquitoes that could have different immune responses than dead mosquitoes. However, we suggest that this had no impact on our conclusions for two reasons. First, the mortality pattern did not follow the melanisation pattern: mortality was highest after injection of two beads, but melanisation was weakest in mosquitoes inoculated with three beads. Second, we performed two tests that included the dead mosquitoes (see Additional file 1). In one, we assumed that the dead mosquitoes also had the weakest immune response, and thus that the lowest melanisation was equal to the lowest melanisation of the group of mosquitoes (according to bead number). In the other, we assumed that the mosquitoes died because of a cost of melanisation, and gave the beads in the dead mosquitoes the strongest melanisation response. In both cases, we reached the same conclusion as with the analyses leaving out the dead mosquitoes.

There are several mechanisms that could potentially lead to our results. Perhaps the most likely is simply due to the need to replenish phenoloxidase reserves [8]. Thus any of the rates in the complex pathway from recognition to melanisation [18] may constrain the production of melanin to the degree that not all beads can be completely melanised. In addition to a constraint on the degree of equilibrium, one might also expect that this constraint may lower the rate of melanisation. Thus, if we had waited longer, the degree of melanisation among beads within mosquitoes would have been less variable. However, melanisation generally takes place within hours [19] and reaches a plateau way within 24 h in mosquitoes inoculated with only one bead [12]. It thus seems highly unlikely that a delay would influence the melanisation observed 48 hours after inoculation.

Mechanisms leading to preferentially targeting some beads over others are less clear. One might suspect that the degree of melanisation within a single mosquito is related to the characteristics of the bead, for example its size. However, we controlled for the size of beads in initial analyses, and it had no influence on the degree of melanisation. One could postulate a positive feedback underlying the regulation of melanisation. Then, the beads that initially (by chance) stimulate a slightly greater response will continue to stimulate the immune response more strongly and reach a greater level of melanisation. However, mechanisms for such a feedback are not known, and indeed, postulated feedback loops are negative [20, 21].

Whatever the mechanism, the fact that the immune response can be overloaded in a way that leads to variability of its outcome within individuals may have considerable implications for the transmission of infectious diseases. Mosquitoes, for example, clear most of the malaria parasites that infect them [22], but, in natural populations, many mosquitoes are unable to clear all of them, and the remaining few are enough to enable effective transmission. The reason for this is unclear. Whereas this ability to clear all parasites is often considered as a measure of qualitative resistance by mosquitoes with an intact immune response, the lack of complete resistance may reflect overloading of mosquitoes with otherwise effective immune responses.

The importance of this variability in the melanisation response could vary according to the type of parasite and some uncertainties remain, some parasites may be killed by the encapsulation, others may be killed by the oxidative cytotoxic compounds produced [23, 24]. So a lower melanisation response may not be sufficient to kill all pathogens. Nevertheless, we certainly expect that the degree of melanisation is essential for ‘large’ parasites such as filarial worms or malaria oocysts.

## Conclusions

The efficacy of the mosquito’s melanisation immune response strongly decreased with the antigen load that stimulates it. Understanding the possible constraints on mosquito immunity by overloading the system, be it with regard to the melanisation response or other immune pathways, may help to understand the evolution of resistance and the transmission of disease.

## Additional file

Additional file 1: Supplementary analysis of immune responses with the dead mosquitoes. (DOCX 87 kb)
