# Supplementary material for: Overloading the immunity of the mosquito Anopheles gambiae with multiple immune challenges
Source: Parasit Vectors. 2016 Apr 14;9:210. doi: 10.1186/s13071-016-1491-8 (PMC4832557; doi:10.1186/s13071-016-1491-8)
Supplement: Additional file 1: — Supplementary analysis of immune responses with the dead mosquitoes. (DOCX 87 kb) [file 13071_2016_1491_MOESM1_ESM.docx]

Additional file 1: supplementary analysis of immune responses with the dead mosquitoes

To rule out the possibility that the bias due to the death of some mosquitoes influenced our results, we redid our analyses with all mosquitoes. As we could not know the melanisation response in the dead mosquitoes, we made two separate assumptions (and two separate tests).

First, we assumed that the dead mosquitoes had the weakest immune response, and thus that in them the most weakly melanised bead had the score of the lowest melanisation within the group of live mosquitoes containing the same number of beads. The analysis of the melanisation response showed no effect of number of beads for the best-melanised beads (F =1.73, P=0.18) and that the melanisation score of the least melanised bead decreased with bead number (F=19.84; P<0.001)

Second, we assumed that the mosquitoes died because of a cost of melanisation, and gave the beads in the dead mosquitoes the strongest melanisation response found in the group of live mosquitoes with the same number of beads. Again, we found no effect of bead number on the best-melanised beads (F =0.46, P=0.63) but a decrease of melanisation for the most weakly melanised beads as (F=4.86; P=0.01).

Thus, for both tests, we obtained the same patterns as in the analyses omitting the dead mosquitoes.
